# Phase I study of MLN8237—investigational Aurora A kinase inhibitor—in relapsed/refractory multiple myeloma, Non-Hodgkin lymphoma and chronic lymphocytic leukemia

**DOI:** 10.1007/s10637-013-0050-9

**Published:** 2013-12-20

**Authors:** Kevin R. Kelly, Thomas C. Shea, André Goy, Jesus G. Berdeja, Craig B. Reeder, Kevin T. McDonagh, Xiaofei Zhou, Hadi Danaee, Hua Liu, Jeffrey A. Ecsedy, Huifeng Niu, Ely Benaim, Swaminathan Padmanabhan Iyer

**Affiliations:** 1 CTRC at the University of Texas Health Science Center at San Antonio, The Institute for Drug Development, San Antonio, TX USA; 2 University of North Carolina, Chapel Hill, NC USA; 3 John Theurer Cancer Center, Hackensack University Medical Center, Hackensack, NJ USA; 4 Sarah Cannon Research Institute, Nashville, TN USA; 5 Division of Hematology and Medical Oncology, Mayo Clinic Arizona, Scottsdale, AZ USA; 6 Markey Cancer Center, University of Kentucky, Lexington, KY USA; 7 Takeda Pharmaceuticals International Company, Cambridge, MA USA; 8 Houston Methodist Cancer Center, 6445 Main Street, Houston, TX 77030 USA

**Keywords:** Phase I-III Leukemia and lymphomas, Novel antitumor agent, Cell cycle mechanisms of anticancer drug action, Aurora A kinase inhibitor, MLN8237

## Abstract

*Purpose* Amplification or over-expression of the mitotic Aurora A kinase (AAK) has been reported in several heme-lymphatic malignancies. MLN8237 (alisertib) is a novel inhibitor of AAK that is being developed for the treatment of advanced malignancies. The objectives of this phase I study were to establish the safety, tolerability, and pharmacokinetic profiles of escalating doses of MLN8237 in patients with relapsed or refractory heme-lymphatic malignancies. *Methods* Sequential cohorts of patients received MLN8237 orally as either a powder-in-capsule (PIC) or enteric-coated tablet (ECT) formulation. Patients received MLN8237 PIC 25–90 mg for 14 or 21 consecutive days plus 14 or 7 days’ rest, respectively, or MLN8237 ECT, at a starting dose of 40 mg/day once-daily (QD) for 14 days plus 14 days’ rest, all in 28-day cycles. Subsequent cohorts received MLN8237 ECT 30–50 mg twice-daily (BID) for 7 days plus 14 days’ rest in 21-day cycles. *Results* Fifty-eight patients were enrolled (PIC *n* = 28, ECT *n* = 30). The most frequent grade ≥3 drug-related toxicities were neutropenia (45 %), thrombocytopenia (28 %), anemia (19 %), and leukopenia (19 %). The maximum tolerated dose on the ECT 7-day schedule was 50 mg BID. The terminal half-life of MLN8237 was approximately 19 h. Six (13 %) patients achieved partial responses and 13 (28 %) stable disease. *Conclusion* The recommended phase II dose of MLN8237 ECT is 50 mg BID for 7 days in 21-day cycles, which is currently being evaluated as a single agent in phase II/III trials in patients with peripheral T-cell lymphoma.

**Electronic supplementary material:**

The online version of this article (doi:10.1007/s10637-013-0050-9) contains supplementary material, which is available to authorized users.

## Introduction

The cell cycle is a tightly regulated process that allows faithful inheritance of genetic material. Several kinase families including the Aurora family of serine/threonine kinases meticulously control the mitotic phase of the cell cycle. In particular Aurora A kinase (AAK) plays an essential role in chromosome alignment, centrosome separation and maturation, mitotic spindle formation, and cytokinesis during mitosis [1–3].

Amplification or over-expression of AAK has been reported in several heme-lymphatic malignancies [4–7], and *Aurora A* may function as an oncogene through the induction of genetic instability and enhanced survival signaling [8, 9]; increased Aurora A expression leads to enhanced cell survival [9]. AAK has also been shown to inhibit the post mitotic checkpoint by targeting p53 for degradation leading to inhibition of p53-mediated apoptosis, and thereby bypassing cell cycle arrest which in turn is associated with genomic instability and oncogenic transformation [10, 11].

The fundamental role of AAK in cell cycle regulation and its aberrant expression in a broad range of malignancies prompted the development of agents that selectively inhibit its activity. Early studies showed that targeted knockdown of AAK led to accumulation of cells in the G_2_/M phase followed by apoptosis and was associated with in vitro and in vivo growth inhibition [12–14]. Inhibition of *AAK* expression has also been shown to disrupt multiple events in mitosis, culminating in monopolar spindle formation, failure of centrosome separation, and incomplete cytokinesis [3, 15].

MLN8237 (alisertib) is an investigational, orally active, selective small molecule inhibitor of AAK that is being investigated for the treatment of both heme-lymphatic malignancies and solid tumors [16]. MLN8237 inhibits AAK with an inhibition constant (Ki) of 0.43 nM [17] and is approximately 200-fold more selective for AAK (IC_50_ = 6.7 nmol/L) than Aurora B kinase (IC_50_ = 1,534 nM/L) in cell-based assays [16]. Moreover, MLN8237 is selective for AAK over other kinases (at least 250-fold more selective in vitro) and receptors [15, 16, 18, 19]. In both preclinical [15, 18, 19] and clinical studies [20, 21], MLN8237 has shown preliminary antitumor activity in heme-lymphatic malignancies. Treatment of multiple myeloma (MM) cells with MLN8237 in vitro resulted in mitotic spindle abnormalities, mitotic accumulation, and apoptosis [15]. In a murine xenograft MM model, tumor burden was significantly reduced (*P* = 0.007) and overall survival was significantly increased (*P* = 0.0044) in animals treated with MLN8237 [15]. Furthermore, targeted inhibition of AAK with MLN8237 showed encouraging antitumor activity in preclinical models of lymphoma [22]. MLN8237 has also been shown to inhibit tumor growth in preclinical models of mantle cell lymphoma, the effect of which was potentiated by docetaxel, and vincristine and rituximab [22, 23]. In two phase I studies in patients with solid tumors, a range of doses and schedules were evaluated and the maximum tolerated dose (MTD) for MLN8237 was determined as 50 mg twice-daily (BID) for 7-day schedule in 21-day cycles [17, 24].

This open-label, phase I study (NCT00697346) was performed to determine the dose-limiting toxicities (DLTs) and MTD, and to evaluate the safety, pharmacokinetics (PKs), and preliminary antitumor activity of MLN8237 in patients with advanced heme-lymphatic malignancies (MM, non-Hodgkin’s lymphoma [NHL], and chronic lymphocytic leukemia [CLL]). Various dosing schedules of two formulations of MLN8237 were assessed in order to determine the optimal strategy to use clinically.

## Patients and methods

### Patients

Eligible patients were aged ≥18 years and had: relapsed or refractory heme-lymphatic malignancy who had failed standard regimens; Eastern Cooperative Oncology Group performance status 0–2; radiographically or clinically evaluable disease; absolute neutrophil count (ANC) ≥1,000/mm^3^ without growth factor support; platelet count ≥50,000/mm^3^ without transfusion requirement; creatinine clearance ≥30 mL/min; total bilirubin ≤1.5 × upper limit of normal (ULN); and aspartate/alanine aminotransferase ≤2.5 × ULN. Patients were excluded if they had received: significant enzyme inducers, enzyme-inducing antiepileptic drugs, or St John’s wort within 14 days; systemic antineoplastic therapy or antineoplastic glucocorticoid treatment within 21 days; radiotherapy (≥25 % of bone marrow) within 42 days; or radio-/toxin-immunoconjugates within 56 days; prior allogeneic bone marrow or other organ transplantation; proton pump inhibitors starting 4 days prior to cycle 1. There was no restriction on the number of prior therapies. Patients were also excluded if they had known gastrointestinal disease that could interfere with oral absorption of MLN8237, conditions that could result in excessive daytime sleepiness, newly diagnosed or uncontrolled cancer-related central nervous system disease, or clinically significant electrocardiogram abnormalities.

### Study design and intervention

This open-label, phase I study was conducted at 9 centers in the USA. The study was carried out in accordance with the Declaration of Helsinki and Good Clinical Practice guidelines. Institutional review boards and local independent ethics committees approved the study and all patients provided their written informed consent for participation.

Patients received escalating doses of MLN8237 as either a powder-in-capsule (PIC) or enteric-coated tablet (ECT) formulation in a standard 3 + 3 design. Initially, only the PIC formulation of MLN8237 was available; the protocol was later modified to include assessment of the ECT formulation. Dosing schedules are summarized in Supplementary Fig. S1 (online only). The PIC formulation starting dose was 25 or 35 mg/day. An initial loading dose was delivered BID on day 1 of each cycle and patients subsequently received MLN8237 once daily (QD) on days 2–14 or 2–21, followed by 14 or 7 days’ rest, respectively, in 28-day cycles. For the ECT formulation, the starting dose was 40 mg/day QD for 14 days followed by 14 days’ rest in 28-day cycles. Subsequent cohorts received MLN8237 ECT BID for 7 days followed by 14 days’ rest in 21-day cycles.

Escalation to the next dose level proceeded only if DLTs were observed in 0/3 or 1/6 patients in cycle 1. If 2 of the first 3–6 patients experienced DLTs at a given dose level, dose escalation was delayed while the available safety/clinical data were assessed and a decision made on how to proceed with a modified dose or schedule. In patients experiencing DLTs, the next cycle of MLN8237 administration was delayed and MLN8237 dose was reduced or discontinued accordingly at the Investigator’s discretion. The use of myeloid growth factors (G-CSF or GM-CSF) or erythropoiesis-stimulating agents was permitted after the DLT evaluation period was completed (cycle 1) and on a case-by-case basis after cycle 2. Treatment continued for up to 12 months, or until disease progression or unacceptable toxicity, with the possibility of treatment extension beyond 12 months if clinical benefit was evident and toxicities were manageable.

### Endpoints

This study had two primary endpoints: to determine the DLTs and MTD of orally administered MLN8237, and to estimate PK parameters during cycle 1. PK parameters included: area under the plasma concentration versus time curve, maximum plasma concentration, time to maximum plasma concentration (T_max_), terminal half-life (t_1/2_), accumulation ratio (R_ac_), and peak/trough ratio. Secondary endpoints were response rate, duration of response, and evaluation of banked tumor tissue samples for AAK protein expression and gene amplification.

### Assessments

#### Safety

Adverse events (AEs) were graded by the National Cancer Institute-Common Terminology Criteria for Adverse Events v3.0 [25]. DLTs were defined as any of the following MLN8237-related AEs occurring during cycle 1: grade 4 neutropenia (ANC <500/mm^3^) at any point during dosing and lasting >7 days during the recovery period, or with fever; grade 4 thrombocytopenia (platelet count <25,000/mm^3^) at any point during dosing and lasting >7 days during the recovery period; grade 3 thrombocytopenia with clinically significant bleeding at any time; platelet count <10,000/mm^3^ at any time; grade ≥3 nausea and/or emesis despite optimal antiemetic prophylaxis; any other grade ≥3 nonhematologic toxicity, with the exception of grade 3 arthralgia/myalgia, any grade of alopecia, or brief (<1 week) grade 3 fatigue; a required rest period of >21 days due to an MLN8237-related toxicity; other grade ≥2 nonhematologic toxicities that, in the opinion of the Investigator, required dose reduction or discontinuation of therapy with MLN8237.

#### Response

Response was evaluated according to the International Working Group criteria for patients with lymphoma [26, 27], the International Myeloma Working Group uniform response criteria for patients with MM [28], and the International Workshop on CLL guidelines [29].

#### Pharmacokinetics

MLN8237 plasma concentrations were measured using a validated liquid chromatography/tandem mass spectrometry assay. Plasma samples were collected in cycle 1 at various time points on the first day (day 1) and last day (day 7, ECT 7-day schedule; day 14, PIC or ECT 14-day schedules; day 21, PIC 21-day schedule) of treatment. PK parameters were calculated by non-compartmental analysis using WinNonlin software (v5.2.1, Pharsight Corporation, Cary, NC).

#### AAK protein expression and gene copy number determination

AAK protein and the phosphorylated Histone H3 (pHH3) at serine 10 (Ser10) expression were determined from available patient archived tumor samples using immunohistochemistry (IHC). *Aurora A* gene and chromosome 20 copy number were determined using fluorescent in situ hybridization (FISH). IHC and FISH were carried out as described in Matulonis UA, et al. 2012 [30].

#### Statistics

Sample size was driven by the dose-escalation scheme and descriptive statistics were employed.

## Results

### Patients

A total of 58 patients were enrolled; 28 to MLN8237 PIC dose levels and 30 to MLN8237 ECT. Patient demographics and baseline characteristics are summarized in Table 1; median age was 61 years (range 27–82), 47 % were male, and 90 % were white. The most common tumor type was NHL (*n* = 36, 62 %), of which the most common subtypes were diffuse large B-cell lymphoma (DLBCL; *n* = 16, 28 %) and follicular lymphoma (FL; *n* = 10, 17 %). Seventy-six percent (*n* = 44) of patients had received ≥3 prior lines of therapy (range 0–17).

**Table 1:** Patient demographics and baseline characteristics

	MLN8237 PIC	MLN8237 ECT	Total
	*n* = 28	*n* = 30	*N* = 58
Median age, years (range)	62 (41–74)	57 (27–82)	61 (27–82)
Male, *n* (%)	14 (50)	13 (43)	27 (47)
Race			
White	24 (86)	28 (93)	52 (90)
Black or African American	3 (11)	1 (3)	4 (7)
Asian	0	1 (3)	1 (2)
Not reported	0	1 (3)	1 (2)
Tumor type, *n* (%)			
Non-Hodgkin lymphoma^a^	18 (64)	18 (60)	36 (62)
Diffuse large B-cell lymphoma	9 (32)	7 (23)	16 (28)
Follicular lymphoma	5 (18)	5 (17)	10 (17)
Mantle-cell lymphoma	2 (7)	0	2 (3)
Peripheral T-cell lymphoma	0	2 (7)^b^	2 (3)
Multiple myeloma	8 (29)	11 (37)	19 (33)
Chronic lymphocytic leukemia/small lymphocytic leukemia	2 (7)	1 (3)	3 (5)
ECOG performance status 0/1/2, *n* (%)	5 (18)/17 (61)/6 (21)	8 (27)/16 (53)/6 (20)	13 (22)/33 (57)/12 (21)
Prior therapy, *n* (%)			
Radiation therapy	11 (39)	14 (47)	25 (43)
ASCT	12 (43)	11 (37)	23 (40)
≥3 prior systemic therapies, *n* (%)	22 (79)	22 (73)	44 (76)
Primary refractory disease	8 (36)	12 (43)	22 (39)

*ASCT* autologous stem-cell transplant; *EBV* Epstein–Barr virus; *ECOG* Eastern Cooperative Oncology Group; *ECT* enteric-coated tablet; *NHL* non-Hodgkin lymphoma; *PIC* powder-in-capsule

^a^Other types of NHL (each *n* = 1) were: angio-immunoblastic T-cell lymphoma; nodal marginal zone B-cell lymphoma; EBV-positive T-cell lymphoma; marginal zone B-cell lymphoma transforming to diffuse large B-cell lymphoma; natural killer/T-cell lymphoma, nasal type; and small cell lymphoma/follicular lymphoma

^b^Both patients had noncutaneous peripheral T-cell lymphoma—not otherwise specified

Patients received a median of 2 treatment cycles (range 1–15); 18 (31 %) patients completed ≥3 cycles, and 8 (14 %) completed ≥6 cycles. Thirty-seven (64 %) patients discontinued due to progressive disease. Treatment is ongoing in 3 (5 %) patients (6, 15, 18 cycles).

### DLTs and determination of MTD

DLTs by MLN8237 dose and schedule are listed in Table 2. On the PIC 21-day QD schedule, 1/6 patients and 2/4 patients in the 25 mg and 35 mg dose levels, respectively, experienced DLTs, including grade 2/3 neutropenia and grade 3 thrombocytopenia. On the PIC 14-day schedule, 0/3 patients who received MLN8237 35 mg reported DLTs, and only 1/6 who received 45 mg experienced a DLT of grade 3 thrombocytopenia resulting in treatment discontinuation. At 65 mg, 0/3 patients experienced a DLT and at 90 mg, 2/2 patients experienced a DLT. The dose was de-escalated to 65 mg and 4 additional patients were enrolled, 2 of whom experienced DLTs (grade 4 neutropenia and grade 4 thrombocytopenia). The MTD on the PIC 14-day schedule was determined to be 45 mg QD.

**Table 2:** DLTs reported in patients receiving MLN8237 treatment

Schedule	Dose level	*N*	Patients with DLT, n	Description of DLTs observed in each patient
PIC 21-day	25 mg QD	6	1	Grade 2 neutropenia, grade 3 thrombocytopenia, grade 3 neutropenia
	35 mg QD	4	2	Grade 3 neutropenia, grade 3 thrombocytopenia resulting in treatment discontinuation Grade 3 neutropenia resulting in treatment discontinuation
PIC 14-day	35 mg QD	3	–	
	45 mg QD	6	1	Grade 3 thrombocytopenia resulting in treatment discontinuation
	65 mg QD	7	2	Grade 4 thrombocytopenia Grade 4 neutropenia
	90 mg QD	2	2	Grade 3 febrile neutropenia Grade 4 thrombocytopenia
ECT 14-day	40 mg QD	6	2	Grade 4 febrile neutropenia Grade 4 bullous dermatitis resulting in treatment discontinuation, grade 4 neutropenia
ECT 7-day	30 mg BID	3	–	
	40 mg BID	9	–	
	50 mg BID	10	1	Grade 4 neutropenia

*BID* twice daily; *DLT* dose-limiting toxicity; *ECT* enteric-coated tablet; *PIC* powder-in-capsule; *QD* once daily

On the ECT 14-day QD schedule, 2/6 patients who received MLN8237 40 mg experienced DLTs (1 patient with grade 4 febrile neutropenia; 1 with grade 4 bullous dermatitis and grade 4 neutropenia), and further dose escalation on this schedule was abandoned. On the ECT 7-day BID schedule, no DLTs were reported in the 30 mg and 40 mg cohorts; 1/10 patients in the 50 mg cohort experienced a DLT of grade 4 neutropenia. The MTD on the ECT 7-day BID schedule was therefore determined as 50 mg BID (ie. 100 mg total dose per day).

### Safety

All 58 patients were included in the safety population. The safety profile for both MLN8237 formulations is summarized in Supplementary Table S1 (online-only). All patients experienced at least 1 treatment-emergent AE, and 90 % reported drug-related AEs. Common all-grade and grade ≥3 drug-related AEs are summarized in Table 3. In total, 35 (60 %) patients (PIC *n* = 14, ECT *n* = 21) reported grade ≥3 drug-related AEs, the most common of which were neutropenia (45 %), and thrombocytopenia (28 %). While frequencies of myelotoxicity somewhat differed between patient groups enrolled to the ECT versus PIC formulations (Table 3), quantitative comparisons are confounded by both the different doses and schedules administered with the two formulations, and potential effects of differing baseline marrow reserves. For example, most patients received the ECT formulation BID for 7 days whereas those receiving MLN8237 PIC received it BID on day 1 only and QD thereafter. The effect of a higher total dose of MLN8237 over a shorter time period probably accounts for the higher rates of neutropenia and thrombocytopenia in the ECT group.

**Table 3:** Most frequent drug-related AEs in ≥10 % (any grade) or ≥5 % (grade ≥3) of patients in the overall population

AE, *n* (%)	MLN8237 PIC		MLN8237 ECT		MLN837 ECT at MTD*			Total	
	*n* = 28		*n* = 30		*n* = 10			*N* = 58	
	All grades	Grade ≥3	All grades	Grade ≥3	All grades	Grade 3	Grade 4	All grades	Grade ≥3
Neutropenia	13 (46)	10 (36)	20 (67)	16 (53)	7 (70)	3 (30)	2 (20)	33 (57)	26 (45)
Thrombocytopenia	11 (39)	8 (29)	13 (43)	8 (27)	5 (50)	–	2 (20)	24 (41)	16 (28)
Diarrhea	8 (29)	–	12 (40)	1 (3)	4 (40)	–	–	20 (34)	1 (2)
Anemia	8 (29)	4 (14)	11 (37)	7 (23)	4 (40)	2 (20)	1 (10)	19 (33)	11 (19)
Fatigue	9 (32)	2 (7)	6 (20)	–	1 (10)	–	–	15 (26)	2 (3)
Vomiting	6 (21)	–	2 (7)	–	–	–	–	8 (14)	–
Alopecia	4 (14)	–	11 (37)	–	4 (40)	–	–	15 (26)	–
Leukopenia	3 (11)	2 (7)	10 (33)	9 (30)	4 (40)	2 (20)	2 (20)	13 (22)	11 (19)
Nausea	9 (32)	–	3 (10)	–	–	–	–	12 (21)	–
Lymphopenia	2 (7)	–	6 (20)	4 (13)	2 (20)	1 (10)	1(10)	8 (14)	4 (7)
Stomatitis	2 (7)	1 (4)	5 (17)	–	1 (10)	–	–	7 (12)	1 (2)
Somnolence	1 (4)	–	5 (17)	–	2 (20)	–	–	6 (10)	–
Decreased WBC count	1 (4)	1 (4)	4 (13)	2 (7)	2 (20)	2 (20)	–	5 (9)	3 (5)
Febrile neutropenia	1 (4)	1 (4)	4 (13)	4 (13)	2 (20)	1 (10)	1 (10)	5 (9)	5 (9)

*MTD, 50 mg BID

*AE* adverse event; *ECT* enteric-coated tablet; *MTD* maximum tolerated dose; *PIC* powder-in-capsule; *WBC* white blood cell

Twenty-eight (48 %) patients (PIC *n* = 12, ECT *n* = 16) experienced serious AEs, the most common of which was febrile neutropenia (PIC *n* = 1, ECT *n* = 4). In total, 9 (16 %) patients (PIC *n* = 4, ECT *n* = 5) discontinued treatment due to AEs of which thrombocytopenia (*n* = 4), neutropenia, bullous dermatitis (*n* = 1), and confusion (*n* = 1) were drug-related.

There were 6 (10 %) on-study deaths (deaths occurring during treatment or within 30 days of last dose); none of which were considered treatment-related. In the PIC population, the 3 reported deaths were due to renal failure, an acute exacerbation of obstructive airways disorder, and progressive lymphoma. In the ECT population, the 3 reported deaths were due to progressive MM (*n* = 2) and hypercalcemia in a patient with DLBCL.

### Pharmacokinetics

**Fig. 1:**
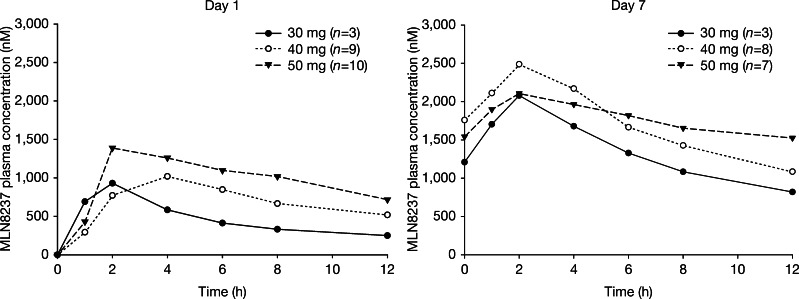
Mean plasma concentration–time profiles of MLN8237 (ECT formulation, 7-day BID schedule) on days 1 and 7 (first dose). *BID* twice daily; *ECT* enteric-coated tablet

Mean plasma concentration–time profiles for MLN8237 ECT BID on days 1 and 7, are shown in Fig. 1. The summary statistics of PK parameters for this dosing regimen are presented in Supplementary Table S2 (online only).

An integrated assessment of the PK parameters was performed across dose cohorts in all 58 patients who received either the PIC or ECT formulation of MLN8237. The validity of this assessment was supported by the lack of a readily apparent nonlinearities in the PK of MLN8237 and the lack of a readily apparent effect of formulation on overall mean apparent oral clearance (CL_ss/_F; mean CL_ss/_F; 4.5 and 5 L/h for MLN8237 PIC or ECT, respectively). MLN8237 absorption was rapid, with an overall median T_max_ of 2 and 2.6 h following oral administration of MLN8237 PIC or ECT, respectively. The overall mean steady-state t_1/2_ following multiple-dose administration was approximately 19 h (range: 10–43 h). The R_ac_ ratio was 2.5 for BID dosing (coefficient of variation [CV %]: 35 %, *n* = 18). Mean CL_ss_/F was 4.8 L/h (CV %: 69 %, *n* = 42).

### Antitumor activity

**Fig. 2:**
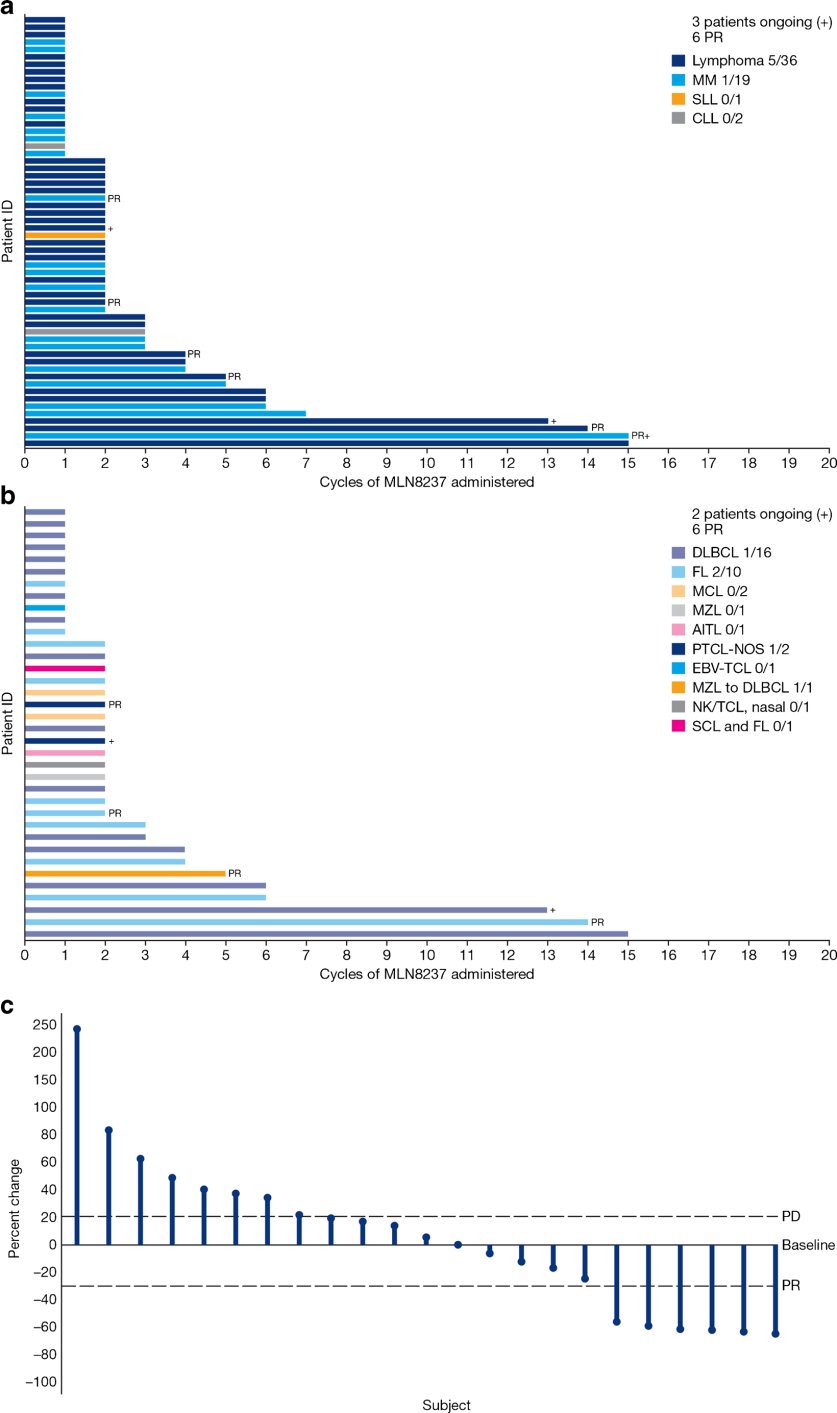
MLN8237 exposure and response in **a** all response-evaluable patients and **b** all response-evaluable patients with lymphomas. Largest percent change in target lesions in patients with lymphoma is shown in panel **c**. *AITL* angioimmunoblastic T-cell Lymphoma; *CLL* chronic lymphocytic leukemia; *DLBCL* diffuse large B-cell lymphoma; *EBV-TCL* Epstein–Barr virus-T cell lymphoma; *FL* follicular lymphoma; *MCL* mantle cell lymphoma; *MM* multiple myeloma; *MZL* marginal zone B-cell lymphoma; *NK/TCL* natural killer/T-cell lymphoma; *PD* progressive disease; *PR* partial response; *PTCL-NOS* peripheral T-cell lymphoma- not otherwise specified; *SCL* small cell lymphoma; *SLL* small lymphocytic leukemia

**Fig. 3:**
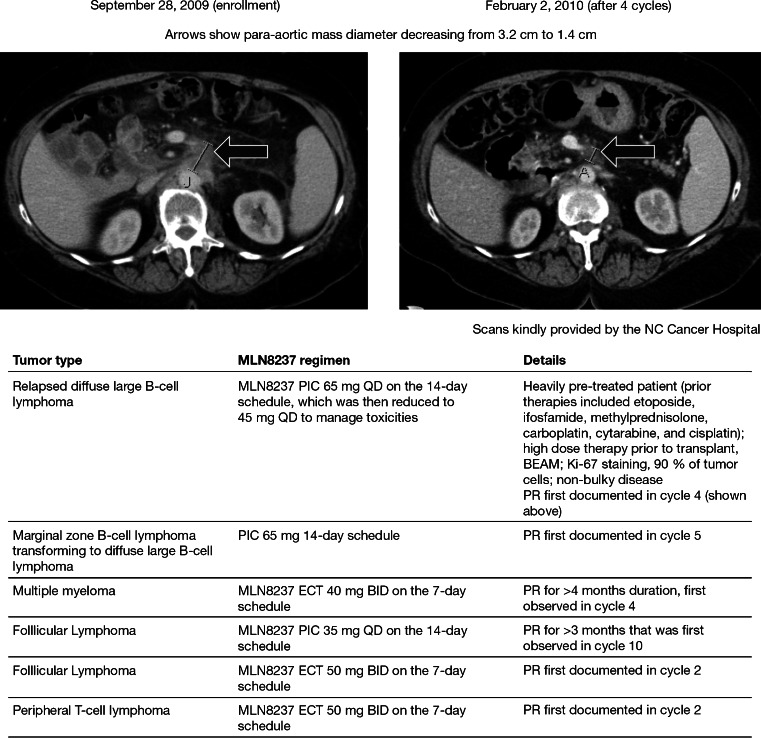
Antitumor activity in a patient with diffuse large B-cell lymphoma receiving 65 mg MLN8237 PIC QD 14-day (*Top*). Tumor type, MLN8237 treatment regimen and additional details for all 6 patients who achieved a PR (*Bottom*). *BEAM* carmustine, etoposide, cytarabine, melphalan; *BID* twice daily; *ECT* enteric coated tablet; *PIC* powder-in-capsule; *PR* partial response; *QD* once daily

Forty-seven patients were response-evaluable; 6 patients had a partial response (PR) and 13 had stable disease (SD) for 1.9–11.0 months (Fig. 2). Scans for a heavily pre-treated patient with relapsed DLBCL who achieved PR are shown in Fig. 3, along with additional details regarding all 6 patients with PR.

### Aurora A protein expression and gene copy number

**Fig. 4:**
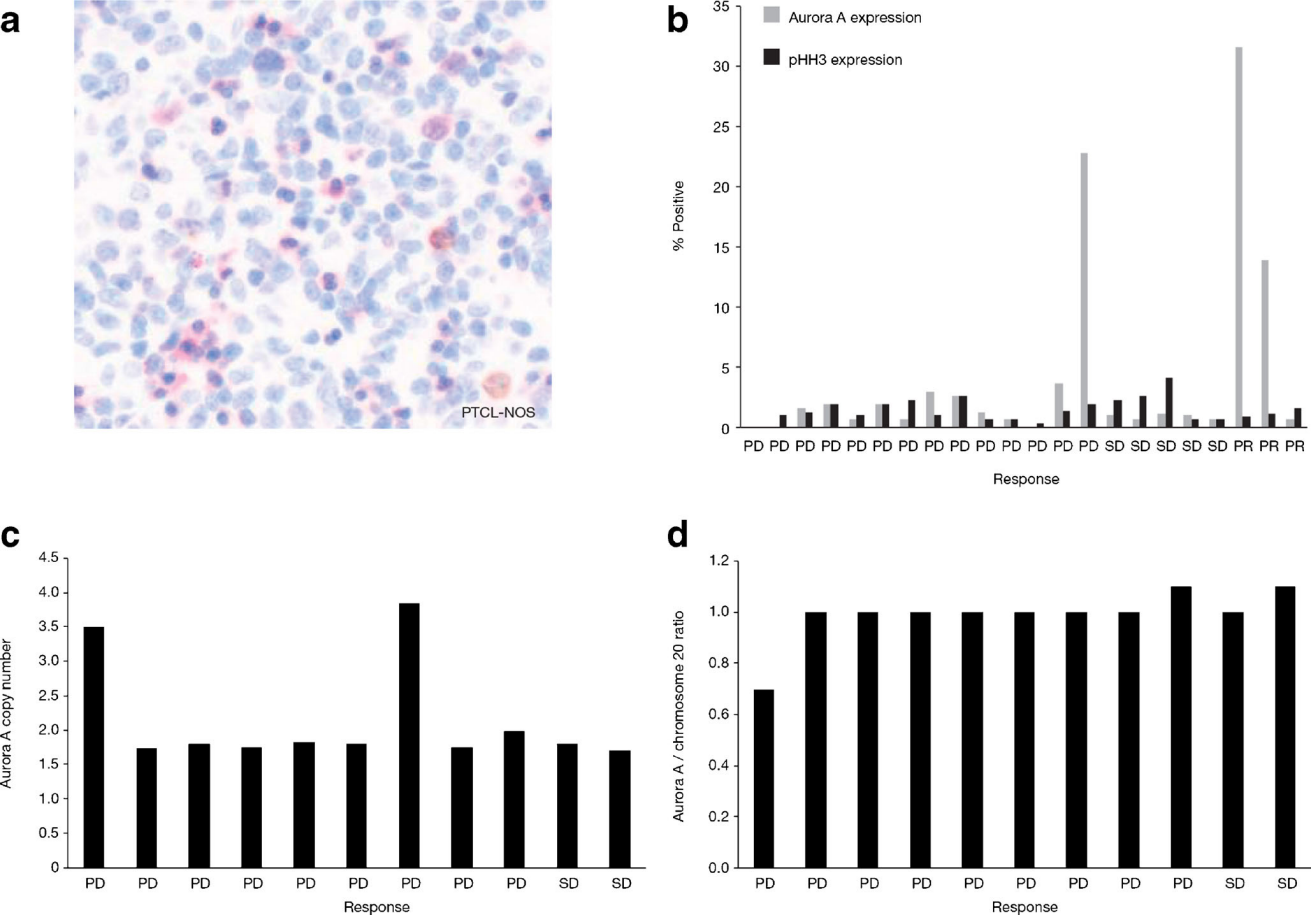
Aurora A and pHH3(Ser10) expression and Aurora A gene copy number inavailable archived tumors. IHC analysis of Aurora A protein expression in a PTCL-NOS sample (**a**). Percentage of cells positive for Aurora A and pHH3 revealed by IHC (**b**). FISH analysis of *Aurora A* gene copy number (**c**). Ratio of *Aurora A* gene copy number relative to chromosome 20 gene copy number (**d**). *FISH* fluorescent in situ hybridization; *IHC* immunohistochemistry; *pHH3(Ser10)* phosphorylated Histone H3 at serine 10; *PD* progressive disease; *PR* partial response; *PTCL-NOS* peripheral T-cell lymphoma- not otherwise specified; *SD* stable disease

AAK and pHH3(Ser10) (used as a marker for the incidence of mitotic cells) protein expression were determined by IHC using available archived tumor biopsies (*n* = 22) collected from patients prior to enrollment in this study (Fig. 4a and b). Of these, 3 response-evaluable patients showed a marked increase in AAK protein levels; 2 of these patients (1 with peripheral T-cell lymphoma- not otherwise specified [PTCL-NOS] and the other with FL) had a PR as best response and the third patient (with EBV-positive T-cell lymphoma), experienced progressive disease. These patients showed variable incidence of mitotic cells (based on pHH3(Ser10) expression levels) and there was no apparent correlation with clinical response (Fig. 4b). *Aurora A* gene and chromosome 20 copy numbers were evaluated by FISH in 11 response-evaluable patients. In 2 of these patients, 1 with T-cell lymphoma and the other with angioimmunoblastic T-cell lymphoma, the analyzed tumors showed an increase in Aurora A gene copy number (Fig. 4c). Interestingly tumor samples from the patient with T-cell lymphoma also showed an increase in Aurora A protein levels. In all analyzed samples the *Aurora A*/chromosome 20 ratio was close to 1, suggesting that the increased Aurora A gene copy number in the 2 patients was due to amplification of chromosome 20 (Fig. 4d).

## Discussion

This was the first phase I study to evaluate the safety and PKs of the investigational AAK inhibitor MLN8237 as PIC or ECT formulations in patients with advanced heme-lymphatic malignancies. Previous studies have shown that doses of MLN8237 25–70 mg for 21 days were associated with predominantly hematologic DLTs [17, 24, 31]. On the other hand, higher daily doses administered over shorter treatment periods such as 7 days have been shown to be generally tolerable [17, 24]. The MTD of single-agent MLN8237 in this patient population with heme-lymphatic malignancies was 45 mg QD on a 14-day schedule for the PIC formulation and 50 mg BID on a 7-day schedule for the ECT formulation. The same MTD for the 7-day BID schedule was defined in two previous phase I studies of MLN8237 PIC [17, 24] and ECT [17] in patients with advanced solid tumors. The ECT formulation has been chosen for future development as it allows MLN8237 (an acidic drug with low aqueous solubility) to bypass stomach acid (gastric pH, 1–4) and delays dissolution until delivery to the small intestine [16, 17].

In the present study, MLN8237 was generally well tolerated. Common side effects included hematologic AEs, gastrointestinal AEs, and alopecia. In general, significant cumulative toxicities were not observed, and the most frequently occurring toxicities were generally reversible in the recovery period between cycles. Although grade 3 or higher neutropenia was reported with high frequency (45 %) across all cohorts, the frequency of grade 3 or higher febrile neutropenia was 9 %. Grade 4 neutropenia was only seen in 1/10 patients treated at the MTD dose of 50 mg BID for the ECT formulation.

The PK properties of MLN8237 in patients with heme-lymphatic malignancies were generally consistent with those observed in a previous study of patients with advanced solid tumors [17]. Based on the integrated PK assessment across dose cohorts in all 58 patients, the range of apparent oral clearance of MLN8237 observed following administration as the ECT formulation was comparable to that observed following administration as PIC, supporting the conclusion of achievement of similar systemic exposures following administration as either the ECT or PIC formulation. This observation is also consistent with results from the bioavailability part of the study, which showed that the bioavailabilities of MLN8237 PIC and ECT were similar [17].

Increased levels of Aurora A protein were observed in 3 response evaluable patients among 22 available archived tumor biopsies. Of note, 2 of these patients had PRs whereas 1 patient experienced progressive disease. In addition, 2 patients had amplified *Aurora A* gene copy number. *Aurora A* amplification in these tumors was most likely due to increased chromosome 20 copy number as the *Aurora A*/chromosome 20 ratio was close to 1.

Aurora A protein overexpression and gene amplification have been linked to the etiology of a variety of cancers, including heme-lymphatic malignancies [4–7]. The number of response evaluable patients with available and informative archived tumor samples was too small to determine if there was a correlation between Aurora A protein expression and gene copy number with MLN8237 response. Identifying biomarkers predictive of response to Aurora kinase inhibitors has been challenging and larger studies will be needed to determine if tumor Aurora A expression or gene amplification correlates with response [32].

MLN8237 has also shown potential antitumor activity in a recent phase II study of single-agent MLN8237 (50 mg BID) in patients with aggressive B- and T-cell NHL [20]. The overall response rate in all 48 treated patients was 27 % (10 % CR and 17 % PR), with 33 % of patients achieving SD. Of particular note, 4/8 patients with T-cell lymphoma had a response [20]. While not a primary focus of the study, the preliminary antitumor activity seen here warrants further investigation. MLN8237 showed signs of antitumor activity as a single agent in patients with advanced heme-lymphatic malignancies including MM, DLBCL, FL, and PTCL. Six (13 %) patients (2 with FL, 1 each with DLBCL, marginal zone B-cell lymphoma transformed to DLBCL, MM, and PTCL) who had each received several prior therapies achieved a PR, and 13 (28 %) achieved SD.

Taken together, these results support further evaluation of the safety and antitumor activity of MLN8237 in NHL and MM in appropriately designed clinical studies. Based on the results of this study and an initial phase II study of MLN8237 in patients with lymphoma [20], a phase II (SWOG S1108; NCT01466881—accrual complete) and a randomized phase III trial of single-agent MLN8237 compared with investigator’s choice of pralatrexate, romidepsin, or gemcitabine in relapsed or refractory PTCL (NCT01482962) is now underway. The rationale for evaluating the clinical activity of MLN8237 in T-cell lymphomas is further supported by recent preclinical studies showing that both AAK and Aurora B kinase are expressed in T-cell lymphoma cell lines [33] and in patient samples from the SWOG S0350 study [34], and that MLN8237 induces endo-reduplication and apoptosis in PTCL cell lines [33]. In addition, based on evidence that MLN8237 combined synergistically with rituximab and vincristine in preclinical models of lymphoma [22], a phase I/II trial of MLN8237 in combination with rituximab and vincristine in patients with relapsed or refractory aggressive B-cell lymphoma is also underway (NCT01397825).

In conclusion, the recommended phase II dose for MLN8237 is 50 mg BID for 7 days followed by a 14-day recovery period, in 21-day cycles. MLN8237 was generally tolerated in these patients with advanced heme-lymphatic malignancies. Preliminary antitumor activity was observed in patients with refractory NHL and MM. MLN8237 continues to be evaluated as a single agent and in combination with other agents in relapsed or refractory NHL.

## Electronic supplementary material

Below is the link to the electronic supplementary material.

ESM 1 (DOC 97 kb)
