# Supplementary material for: Phase I study of MLN8237—investigational Aurora A kinase inhibitor—in relapsed/refractory multiple myeloma, Non-Hodgkin lymphoma and chronic lymphocytic leukemia
Source: Invest New Drugs. 2013 Dec 20;32(3):489–99. doi: 10.1007/s10637-013-0050-9 (PMC4045308; doi:10.1007/s10637-013-0050-9)
Supplement: Supplementary file 1 — (DOC 97 kb) [file 10637_2013_50_MOESM1_ESM.doc]

***Investigational New Drugs Supplementary information***

**Phase I Study of MLN8237 – Investigational Aurora A Kinase Inhibitor – in Relapsed/Refractory Multiple Myeloma, Non-Hodgkin Lymphoma and Chronic Lymphocytic Leukemia**

**Authors:**

Kevin R. Kelly^1^, Thomas C. Shea^2^, André Goy^3^, Jesus G. Berdeja^4^, Craig B. Reeder^5^, Kevin T. McDonagh^6^, Xiaofei Zhou^7^, Hadi Danaee^7^, Hua Liu^7^, Jeffrey A Ecsedy^7^, Huifeng Niu^7^, Ely Benaim^7^, and Swaminathan Padmanabhan Iyer^8^

**Author affiliations:**

^1^CTRC at the University of Texas Health Science Center at San Antonio, The Institute for Drug Development, San Antonio, TX, USA; ^2^ University of North Carolina, Chapel Hill, NC, USA; ^3^John Theurer Cancer Center, Hackensack University Medical Center, Hackensack, NJ, USA; ^4^Sarah Cannon Research Institute, Nashville, TN, USA; ^5^Division of Hematology and Medical Oncology, Mayo Clinic Arizona, Scottsdale, AZ, USA; ^6^Markey Cancer Center, University of Kentucky, Lexington, KY, USA; ^7^Millennium: The Takeda Oncology Company, Cambridge, MA, USA; ^8^The Houston Methodist Cancer Center, Houston, TX, USA

**Corresponding author:**

Swaminathan Padmanabhan Iyer, MD

6445 Main Street., Houston, TX 77030, USA

Telephone: +1 713-441-0687

Fax: +1 713-793-1642

E-mail:spiyer@houstonmethodist.org

**Supplementary Material**

**Supplementary Table S1** Overview of the safety profile of MLN8237 as either a PIC or ECT formulation

| ***n* (%)** | **MLN8237 PIC**  ***n*=28** | **MLN8237 ECT**  ***n*=30** | **Total**  ***N*=58** |
| --- | --- | --- | --- |
| Any treatment-emergent AE | 28 (100) | 30 (100) | 58 (100) |
| Any treatment-emergent grade ≥3 AE | 21 (75) | 25 (83) | 46 (79) |
| Any drug-related AE | 25 (89) | 27 (90) | 52 (90) |
| Any drug-related grade ≥3 AE | 14 (50) | 21 (70) | 35 (60) |
| Any treatment-emergent serious AE | 12 (43) | 16 (53) | 28 (48) |
| Any AE resulting in discontinuation | 4 (14) | 5 (17) | 9 (16) |
| On-study deaths | 3 (11) | 3 (10) | 6 (10) |

AE, adverse event; PIC, powder-in-capsule; ECT, enteric-coated tablet

**Supplementary Table S2** Summary of PK parameters for MLN8237 (BID ECT 7-day schedule; first dose)

| Dose (mg) | Day | *N* | Geometric mean  C_max_ (nM)  (CV, %) | Median T_max_, h  (range) | Geometric mean  AUC_0–tlast_,  nM*h  (CV, %) | Mean t_1/2_, hr (sd) | Maen R_ac_  (sd) | Mean  peak/trough ratio (sd) | Geometric mean CL_ss_/F, L/h  (CV, %) |
| --- | --- | --- | --- | --- | --- | --- | --- | --- | --- |
| 30 | 1 | 3 | 886 (39.7) | 2.0 (2.0–2.0) | 5518 (18.3) | NA | NA | NA | NA |
|  | 7 | 3 | 2025 (29.6) | 2.0 (2.0–2.0) | 16024 (20.3) | 13.3* | 2.9 (0.5) | 2.5 (0.6) | 3.7 (0.7) |
| 40 | 1 | 9 | 1114 (37.1) | 2.2 (2.0–6.0) | 7095 (42.5) | NA | NA | NA | NA |
|  | 7 | 8 | 2586 (35.7) | 2.0 (1.0–3.6) | 18624 (27.3) | 19.9 (10.7)** | 2.8 (1.0) ^†^ | 2.4 (0.5) ^†^ | 4.4 (1.9) ^†^ |
| 50 | 1 | 10 | 1531 (58.4) | 2.0 (2.0–8.0) | 9732 (48.5) ^‡^ | NA | NA | NA | NA |
|  | 7 | 7 | 2058 (44.6) | 2.0 (1.3–6.0) | 17914 (48.6) | 18.4 (13.9)^§^ | 2.3 (0.9)^‖‖^ | 2.5 (2.2) | 6.7 (5.6) |

**n*=1, ** *n*=4, ^†^ *n*=7, ^‡^ *n*=9, ^§^ *n*=5, ^‖‖^ *n*=6.

AUC_0–tlast_, area under the plasma concentration versus time curve; BID, twice daily; CL_ss_/F, overall mean apparent oral clearance; C_max_, maximum plasma concentration; CV, coefficient of variation; PK, pharmacokinetic; NA, not applicable; R_ac_, accumulation ratio; sd, standard deviation; t_1/2_, terminal half-life; T_max_, time to maximum plasma concentration.

**Supplementary Fig. S1** Treatment schedules for MLN8237 in the PIC and ECT formulations

**
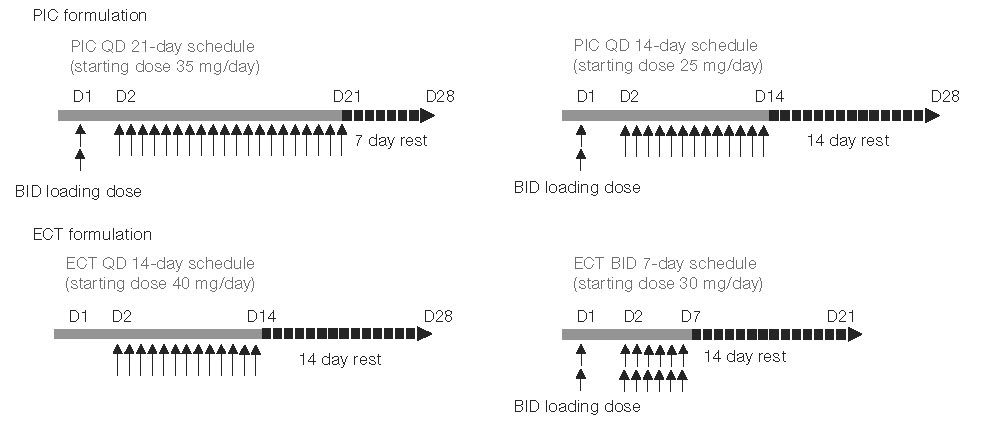
**

BID, twice-daily; D, day; ECT, enteric-coated tablet; PIC, powder-in-capsule; QD, once-daily.
